# Assessment of genomic imprinting of *SLC38A4*, *NNAT*, *NAP1L5*, and *H19 *in cattle

**DOI:** 10.1186/1471-2156-7-49

**Published:** 2006-10-25

**Authors:** Ismail Zaitoun, Hasan Khatib

**Affiliations:** 1 Department of Dairy Science, University of Wisconsin-Madison, 1675 Observatory Dr., Madison, WI 53706, USA

## Abstract

**Background:**

At present, few imprinted genes have been reported in cattle compared to human and mouse. Comparative expression analysis and imprinting status are powerful tools for investigating the biological significance of genomic imprinting and studying the regulation mechanisms of imprinted genes. The objective of this study was to assess the imprinting status and pattern of expression of the *SLC38A4*, *NNAT*, *NAP1L5*, and *H19 *genes in bovine tissues.

**Results:**

A polymorphism-based approach was used to assess the imprinting status of four bovine genes in a total of 75 tissue types obtained from 12 fetuses and their dams. In contrast to mouse *Slc38a4*, which is imprinted in a tissue-specific manner, we found that *SLC38A4 *is not imprinted in cattle, and we found it expressed in all adult tissues examined. Two single nucleotide polymorphisms (SNPs) were identified in *NNAT *and used to distinguish between monoallelic and biallelic expression in fetal and adult tissues. The two transcripts of *NNAT *showed paternal expression like their orthologues in human and mouse. However, in contrast to human and mouse, *NNAT *was expressed in a wide range of tissues, both fetal and adult. Expression analysis of *NAP1L5 *in five heterozygous fetuses showed that the gene was paternally expressed in all examined tissues, in contrast to mouse where imprinting is tissue-specific. *H19 *was found to be maternally expressed like its orthologues in human, sheep, and mouse.

**Conclusion:**

This is the first report on the imprinting status of *SLC38A4*, *NAP1L5*, and on the expression patterns of the two transcripts of *NNAT *in cattle. It is of interest that the imprinting of *NAP1L5*, *NNAT*, and *H19 *appears to be conserved between mouse and cow, although the tissue distribution of expression differs. In contrast, the imprinting of *SLC38A4 *appears to be species-specific.

## Background

Based on data from the Catalogue of Imprinted Genes [1], the number of known imprinted genes reported for cattle is small compared to human and mouse. At present, few imprinted genes have been reported in cattle, among them are *IGF2R *[2]; *XIST*, *IGF2*, *GTL2 *[3]; *PEG3 *[4]; *NESP55 *[5]; *H19 *[6]; and *NNAT *[7]. With progress of the bovine genome project, the available sequence data will accelerate the discovery of other imprinted genes. Most of the genes examined for imprinting in human and mouse have shown conservation; 29 out of 41 imprinted genes in human have been found to be imprinted in the mouse [8]. Recently, we reported that the *COPG2*, *DCN*, and *SDHD *genes are not imprinted in cattle while their orthologues are imprinted in mouse or human [9]. The aim of this study was to investigate the imprinting status of bovine *SLC38A4*, *NNAT*, *NAP1L5*, and *H19 *and analyze their patterns of expression in fetal and adult tissues.

The *SLC38A4 *(solute carrier family 38, member 4) gene, also named *ATA3*, is a member of the amino acid transport system A gene family that mediates the uptake of short-chain, neutral, aliphatic amino acids [10]. In a search for novel imprinted genes in mouse, using differential expression between parthenogenetic and androgenetic embryos, Mizuno et al. [11] found that *Slc38A4 *is paternally expressed in a wide range of fetal tissues except the liver and viscera. In a different mouse study, Smith et al. [12] found that *Slc38A4 *is imprinted in placenta and is biallelically expressed in adult liver tissue.

The neuronatin gene (*NNAT*) was originally identified in brains of neonatal rats [13]. On Northern blot analysis, it was shown that *NNAT *is highly expressed in fetal brain of human and rat but is downregulated in adult human brain and shows low expression in adult rat brain [13]. The brain-specific pattern of expression suggests the involvement of *NNAT *in brain development [14]. The human gene contains three exons and two introns that encode two alternatively spliced transcripts: α, which includes all three exons and β, which skips exon 2 [15]. Evans et al. [16] reported that human *NNAT *is paternally expressed and is located within the intron of the *BLCAP *gene which is not imprinted. Kagitani et al. [17] showed that mouse *Nnat *has four alternatively spliced transcripts, all of which are expressed from the paternal allele.

Nucleosome assembly protein 1-like 5 (*NAP1L5*) was first identified in a search for imprinted genes in mouse using methylation-sensitive representational difference analysis in parthenogenetic embryos [12]. The function of *NAP1L5 *is not known, but its protein shows homology to nucleosome assembly proteins (NAPs); NAP1 is involved in translocation of histones from the cytoplasm into the nucleus and in cell cycle regulation [18]. Smith et al. [12] reported that *Nap1l5 *is paternally expressed in brain and adrenal glands of adult mice. In addition, they found that the entire gene is located within the intron of the *Herc3 *gene, which is not imprinted [12].

The *H19 *gene was originally discovered in the mouse in a search for fetal cDNA clones under the regulation of the murine *raf *and *Rif *genes [19]. It has been proposed that *H19 *is associated with embryogenesis and fetal growth in mouse [19], human [20], and sheep [21]. Although the imprinting and gene expression of *H19 *have been well studied in the mouse and human, knowledge of the expression pattern in cattle has been limited to only one study on the imprinting status in two newborn calves [6].

This is the first report on the imprinting status of *SLC38A4*, *NAP1L5*, and on the expression patterns of the two transcripts of *NNAT *in cattle. In addition, we report the expression patterns of these genes in a wide range of fetal and adult tissues.

## Results and discussion

### Expression analysis of *SLC38A4*

**Figure 1:**
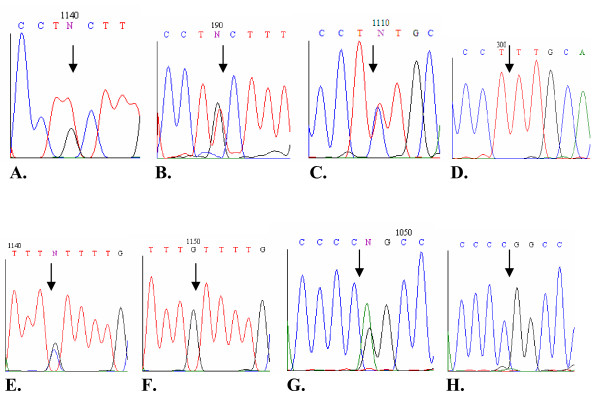
**Sequence analysis of genomic DNA and cDNA for *SLC38A4 *(A, B), *NNAT *(C, D), *NAP1L5 *(E, F), and *H19 *(G, H) genes**. Arrows point to SNP site. **A, C, E, and G**, sequence analysis of genomic DNA shows heterozygosity at positions 9188, 11761, 1024, and 1889 of *SLC38A4*, *NNAT*, *NAP1L5*, and *H19*, respectively. **B**, analysis of cDNA shows biallelic expression of C and T alleles at SNP 9188. **D, F, and H**, for the imprinted genes *NNAT*, *NAP1L5*, and *H19*, monoallelic expression of alleles T, G, and G, is shown.

This is the first report on the imprinting status of *SLC38A4 *in cattle. A search for polymorphisms in the coding sequence of the bovine *SLC38A4 *gene in a total of 19 individuals revealed one SNP (T/G) at position 9188 in six individuals. To analyze the expression status of *SLC38A4 *in these heterozygous individuals, SNP 9188 was used to distinguish between monoallelic and biallelic expression, by comparing the sequenced RT-PCR products with the sequenced genomic DNA PCR products. An example of biallelic expression of *SLC38A4 *is shown in Figure 1B, along with the genomic DNA sequence (Figure 1A) at SNP 9188.

For fetuses 2, 4, and 10, sequencing of RT-PCR products obtained from the kidney, spleen, cartilage, pancreas, heart, brain, lung, eye, and testis revealed biallelic expression (T/G). For dams 1, 7, and 15, biallelic expression was observed in the endometrium, ovary, oocytes, caruncle, liver, spleen, lung, and skeletal muscle tissues (Table 2). No monoallelic expression was observed.

**Table 1:** Primer sequences and products amplified from the bovine *SLC38A4*, *NNAT*, *NAP1L5*, *H19*, and b-actin genes

**Primer**	**Sequence**	**Product size (bp) and type**	**Number of individuals tested**
SLC-Fg	TTC ATT CAC TTT GGC TCC ATG CAG C	355; DNA	19
SLC-Rg	AAT CAT GCT GCT TGC TGT GG		
SLC-F	CCG CTG GTA TAA CCA AGG TAA	354; cDNA	
SLC-R	AAT CAT GCT GCT TGC TGT GG		
SLC.EXT	ACC TAG TTT TTC ATA AAT TAA AGA CCC TCC T	31; primer extension	

NNAT-Fg	CTA AGT TGT GGG TCC AAT CAG CT	695; DNA	11
NNAT-Rg	TGT AGT TGT CTG GAT CTC TGT GGT G		
NNAT-FL^a^	TTT CCG CGT GCT GCT GCA GGT GTT CCT	453; cDNA	
NNAT-R	TCC CCC TAA GCC CCG TTC CT		
NNAT-FS^b^	TTT CCG CGT GCT GCT GCA GGT GTT CAG GT	370; cDNA	
NNAT-R	TCC CCC TAA GCC CCG TTC CT		
NNAT.EXT	GAC AAT GAC GAC AAC AAG AGA TCC CTT CCC CAC CCC T	37; primer extension	

NAP1L5-F	GTG TGC ATG GAC CTT AGA GG	730; DNA,	13
NAP1L5-R	TTG TCA TGA TCT CCA GCA CC	cDNA	
NAP1L.EXT	TTC TCA ATG CCG AAT TCT TCC ATT T	25; primer extension	

H19-JY511	GAC CTA AAG GAA CGG ACG AC	192; DNA	40
H19-JY318	TC CTG AGC AAA GGA TAG CAGA		
H19F	GTG CCT CTG AGC TCG GAA CG	580; cDNA	
H19R	CTC CTG AGC AAA GGA TAG CAGA		
H19.EXT	CCG CGG CGA CAC CCA CCC C	19; primer extension	

b-actin F	CAGCACAATGAAGATCAAGATCATC	191; cDNA	N/A
b-actin R	AAAGGGTGTAACGCAGCTAACAGT		

**Table 2:** Expression analysis of the transcripts of the bovine *SLC38A4 *gene in heterozygous individuals

**Individual**	**Tissue**	**Alleles expressed at position 9188^a^**
Fetus 2	Kidney, spleen, cartilage, pancreas	T/G
Fetus 4	Kidney, heart, brain, lung	T/G
Fetus 10	Pancreas, eye, testis, lung	T/G
Dam 1	Endometrium, ovary, oocytes	T/G
Dam 7	Liver, spleen, caruncle	T/G
Dam 15	Lung, muscle, caruncle	T/G

^a^SNP position based on the numbering in GenBank accession number NW_391237

**Figure 2:**
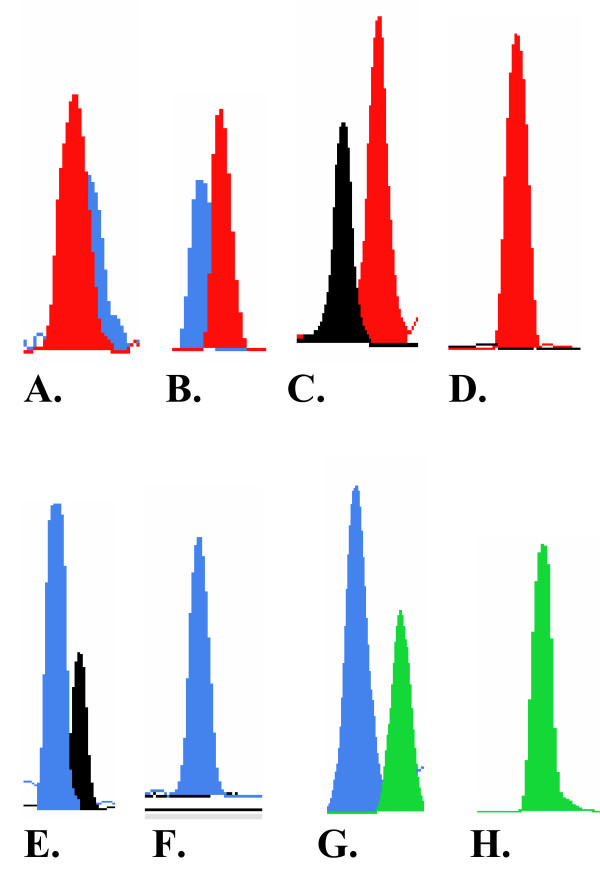
**Analysis of genomic DNA and cDNA for *SLC38A4 *(A, B), *NNAT *(C, D), *NAP1L5 *(E, F), and *H19 *(G, H) genes using the primer extension method**. **A, C, E, and G**, show two peaks in genomic DNA obtained from heterozygous individuals. **B**, primer extension analysis revealed two peaks (representing biallelic expression of *SLC38A4*) in cDNA products obtained from a wide range of tissues. **D, F, and H**, primer extension analysis shows monoallelic expression for *NNAT*, *NAP1L5*, and *H19 *respectively.

Biallelic expression of *SLC38A4 *was confirmed using primer extension reactions of several representative RT-PCR products. This method has been used to assay allelic variation in gene expression [22,23] and to verify the imprinting status of cattle genes [24]. Figures 2A and 2B show examples of primer extension analysis for the *SLC38A4 *gene. RT-PCR products showed two peaks (representing T and G alleles) at position 9188. Thus, based on the expression analysis in fetal and adult tissues, *SLC38A4 *is not imprinted in cattle.

In contrast, Mizuno et al. [11] reported that the murine *Slc38A4 *gene is imprinted in a tissue-specific manner. In a search for differentially expressed genes between parthenogenetic and androgenetic embryos, they found that *Slc38A4 *is paternally expressed in all embryonic tissues but the liver and viscera. In a different mouse study, Smith et al. [12] reported that the murine *Slc38A4 *is paternally expressed in placenta and biallelically expressed in adult liver. Moreover, using Northern blot hybridization, Smith et al. [12] found that *Slc38A4 *is highly expressed in liver and placenta with decreased or no expression in other adult tissues. Also, using Northern blot analysis, Sugawara et al. [10] found that rat *Slc38A4 *is highly expressed in liver with lower expression levels in skeletal muscle. In contrast to rodent studies, we found that the bovine *SLC38A4 *gene was expressed in all adult tissues examined (Table 2). For fetal tissues, our results showed an expression-level pattern similar to that found in the mouse by Mizuno et al. [11].

### Imprinting and expression of *NNAT*

The in silico search for polymorphisms in the bovine *NNAT *gene revealed two SNPs, at positions 11738 (C/T) and 11761 (G/T) in exon 3. Primers NNAT-Fg/NNAT-Rg were used to amplify genomic DNA from a total of 11 fetuses and dams. Sequencing of PCR products confirmed the presence of these two SNPs in three fetuses and in one dam. A sequencing-based approach was used to analyze the imprinting status of the *NNAT *gene in the heterozygous individuals. Primers NNAT-FL/NNAT-R were used to amplify 453 bp of cDNA from the large transcript (α) of *NNAT *which includes exons 1, 2, and 3. Primers NNAT-FS/NNAT-R were used to amplify 370 bp of cDNA from transcript β which includes exons 1 and 3. To ensure transcript-specific amplification of transcript α, the first 19 nucleotides of primer NNAT-FL were designed in exon 1 and the last eight nucleotides were designed in exon 2. For transcript-specific amplification of transcript β, the first 19 nucleotides of primer NNAT-FS were designed in exon 1 and the last ten nucleotides were designed in exon 3. In addition, primers spanning more than one exon would exclude the possibility of DNA contamination in the RT-PCR reactions.

Table 3 shows the imprinting status of the two transcripts of *NNAT *in tissues from heterozygous individuals. Sequencing of RT-PCR products amplified from tissues of fetuses 1, 3, 17, and dam 1 revealed monoallelic expression of the two transcripts at positions 11738 and 11761 in all examined tissues. As shown in Figure 1D, *NNAT *transcripts are monoallelically expressed. The monoallelic expression was also confirmed by use of primer extension analysis. Figure 2C shows two peaks representing C and T alleles in genomic DNA of a heterozygous individual, whereas Figure 2D shows the one peak representing allele T at position 11738 of *NNAT *cDNA. Thus, the results of the primer extension analysis confirm the results obtained by the sequencing-based approach shown in Figure 1 and Table 3.

**Table 3:** Expression analysis of the transcripts of the bovine *NNAT *gene in heterozygous individuals

**Individual**	**Tissue(s)**	**Allele(s) expressed^a^**	
		**11738**	**11761**
Fetus 1^b^	Lung, brain, liver, kidney, muscle	T	T
Fetus 3^b^	Ovary, pituitary, mammary gland, brain	T	T
Fetus 17^b^	Eye, intestine, brain	T	T
Dam 1^c^	Endometrium	C	G
Fetus 1^c^	Lung, brain, liver, kidney, muscle	T	T
Fetus 3^c^	Ovary, pituitary, mammary gland, brain, lung, liver	T	T
Fetus 17^c^	Eye, intestine, brain, spleen, heart	T	T

^a^11738 and 11761 are positions of the SNPs, C/T and G/T, respectively, in the bovine *NNAT *gene (accession number NW_275903); ^b^transcript α of *NNAT *which includes exons 1, 2, and 3; ^c^transcript β of *NNAT *which includes exons 1 and 3.

For the human gene, Evans et al. [16] utilized a SNP in intron 1 to analyze the expression status of *NNAT *in fetal brains. Using unspliced nuclear RNA sequencing, they showed that both α and β transcripts are paternally expressed [16]. Kagitani et al. [17] identified four transcripts of *Nnat *in the mouse, of which transcripts 2 and 3 correspond to human transcripts α and β. They found that all four alternatively spliced isoforms were paternally expressed in mouse embryos. In cattle, using differential expression between in vitro-fertilized and parthenogenetic embryos, Ruddock et al. [7] reported that *NNAT *is imprinted during preimplantation developmental stages. In this study, we applied the sequence-based approach and investigated the expression status of the two transcripts of *NNAT *in both fetal and maternal tissues. Like its orthologues in human and mouse, *NNAT *is imprinted in cattle.

**Figure 3:**
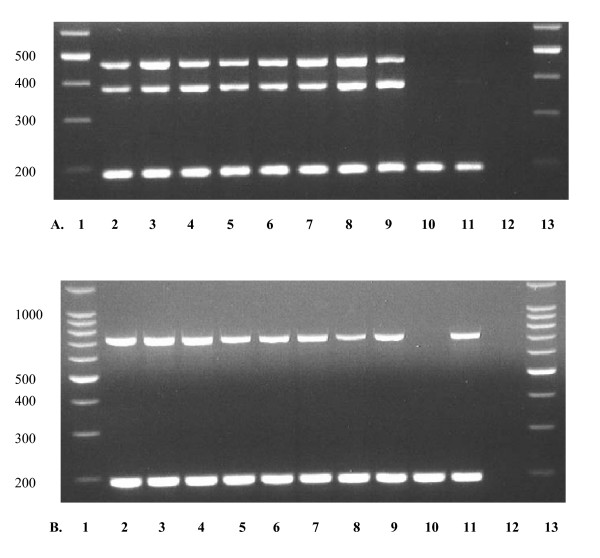
**Expression of *NNAT *and *NAP1L5 *transcripts obtained from cattle adult tissues and analyzed by RT-PCR**. **A**, Expression analysis of *NNAT *transcripts. Lanes 1 and 13 are a 100-bp ladder marker. Lanes 2–11 are RT-PCR products obtained from adult ovary, endometrium, caruncle, heart, muscle, lung, liver, kidney, pancreas, and spleen, respectively. Lane 12 is a negative control. The sizes of RT-PCR products were 453 bp for transcript α (top bands), 370 bp for transcript β (middle bands), and 191 bp for b-actin (bottom bands). **B**, *NAP1L5 *transcripts amplified with NAP1L5-F/NAP1L5R. Lanes 1 and 13 are a 100-bp ladder marker. Lanes 2–11 are RT-PCR products obtained from adult ovary, endometrium, caruncle, heart, muscle, lung, liver, kidney, pancreas, and spleen, respectively. Lane 12 is a negative control. The bottom bands are RT-PCR products of b-actin.

Of considerable interest was the observation that rat *Nnat *was highly expressed in fetal brain but not in heart, liver, kidney and other tissues [14]. The same investigators also observed minimal expression levels in adult male and female brains. Evans et al. [16] reported that human *NNAT *was expressed in fetal brain but not in fetal adrenal gland, gut, heart, kidney, liver, spleen, muscle, placenta, or spleen. In contrast to findings in rat and human, we show in this study that bovine *NNAT *is expressed in a wide range of fetal tissues including lung, liver, kidney, muscle, ovary, eye, and intestine, in addition to pituitary and brain (Table 3). Moreover, for adult cow, *NNAT *transcripts were detectable in ovary, endometrium, caruncle, lung, liver, kidney, heart, and muscle (Fig. 3A), in contrast to the loss of expression in adult rat tissues observed by Joseph et al. [14]. *NNAT *expression was not detected in pancreas or spleen (Fig. 3A).

### Imprinting and expression of *NAP1L5*

The sequencing of four RNA pools revealed one SNP at position 1024 in the bovine *NAP1L5 *gene. To identify informative individuals, DNA from 11 fetuses and dams was amplified using primers NAP1L5-F/NAP1L5-R. Sequencing of PCR products revealed five heterozygous individuals. Table 4 shows the expression analysis of *NAP1L5 *in tissues obtained from these individuals. Tissues of fetuses 1, 2, 8, and 14 expressed the G allele while all tissues of fetus 12 expressed the C allele. Figure 1E shows sequence analysis of genomic DNA obtained from an individual heterozygous for SNP C/G at position 1024. Figure 1F presents an example of monoallelic expression of allele C of *NAP1L5*. The genotyping and sequencing of amplified genomic DNA of the dams of fetuses 1, 8, 12, and 14 showed that those dams were heterozygous, so the parental origin of the imprinted allele could not be determined in these fetuses. The dam of fetus 2 was homozygous for allele C so, for this fetus, *NAP1L5 *expression was clearly paternal.

**Table 4:** Expression analysis of the transcripts of the bovine *NAP1L5 *gene in heterozygous individuals

**Individual**	**Tissues**	**Allele expressed^a^**
Fetus 1	Brain, liver, kidney, muscle	G
Fetus 2	Mammary gland, spleen, cartilage, pancreas, liver, muscle, kidney, heart, hypothalamus, ovary, lung, brain	G
Fetus 8	Brain, intestine, eye, pancreas, heart, mammary gland, muscle, ovary, kidney, cartilage, liver, lung	G
Fetus 12	Kidney, spleen, heart, liver, muscle, brain	C
Fetus 14	Brain, muscle, spleen, liver, lung, mammary gland, cotyledon, eye, intestine, heart, kidney	G

^a^expression of SNP alleles at position 1024 based on the numbering in GenBank accession number XM_585294

The imprinting status of *NAP1L5 *was also confirmed using primer extension reactions of several RT-PCR products obtained from different tissues. Figure 2E shows two peaks – obtained from PCR products – representing the C and G alleles of a heterozygous individual. Figure 2F shows monoallelic expression of allele C obtained from RT-PCR products.

No human data are available on imprinting status of the homologous gene. Smith and colleagues [12] found in the mouse, that *Nap1l5 *is paternally expressed in brain and adrenal glands, similar to the paternal expression that we found with the bovine gene. Smith et al. [12] observed no evidence of *Nap1l5 *expression in adult mouse heart, kidney, spleen, thymus, liver, or lung. In contrast to mouse, the bovine gene showed strong expression in adult ovary, endometrium, caruncle, lung, liver, spleen, kidney, heart, and muscle tissues (Fig. 3B). We did not detect *NAP1L5 *expression in adult pancreas (Fig. 3B). In the fetuses, *NAP1L5 *was highly expressed in 15 different tissue types including brain, liver, kidney, muscle, mammary gland, spleen, heart, hypothalamus, ovary, lung, intestine, eye, pancreas, cartilage, and cotyledon (Table 4). Thus, for pancreas, *NAP1L5 *is downregulated in adult tissues. The high expression level of *NAP1L5 *in a wide range of fetal tissues suggests this gene has possible roles in fetal growth and development. This is the first report on the expression of *NAP1L5 *in fetuses and adults. It would be of interest to further investigate this gene in other mammalian species to shed more light on its expression and function.

It is worth noting that *NAP1L5 *is an intron-less gene that is located in the intron of the *HERC3 *gene [12]. When studying gene expression of a one-exon gene, it is critical to remove residuals of DNA from the RNA samples. To exclude the possibility of DNA contamination in the RT-PCR amplifications, we performed the following steps: 1) Total RNA was treated with DNase I in two rounds with longer incubation times than recommended by the manufacturer. 2) After each round of total RNA extraction, we used RNA samples as template for PCR amplifications with 3 or 4 different pairs of primers (data not shown). DNase I treatments were repeated until the complete digestion of the DNA in the RNA samples was assured. For the other genes, primers were designed to amplify fragments spanning more than one exon.

### Imprinting of *H19*

Primers JY511 and JY318 [6] were used to amplify genomic DNA from 40 fetuses and dams. Sequencing of PCR products revealed four fetuses and three dams heterozygous for a SNP (A/G) at position 1889. To analyze the expression status of *H19*, primers H19F/H19R were used to amplify total RNA from a total of 58 fetal and adult tissues. Table 5 shows the expression analysis of *H19 *in heterozygous individuals. For fetus 9 and dam 6, all examined tissues showed monoallelic expression of the G allele, whereas tissues of all other fetuses and dams showed monoallelic expression of the A allele (Table 5). The genotyping of dams of fetuses 7 and 9, using PCR of genomic DNA, showed that those dams were heterozygous, so the parental origin of the imprinted allele could not be determined in these fetuses. The dams of fetuses 5 and 16 were homozygous for allele G so, for these fetuses, *H19 *expression was clearly maternal. The allelic expression of *H19 *was examined in the DNA and cDNA obtained from heterozygous individuals using both the sequencing-based and primer-extension approaches (Fig. 1G, 1H; Fig. 2G, 2H).

**Table 5:** Expression analysis of the transcripts of the bovine *H19 *gene in heterozygous individuals

**Individual**	**Tissue(s)**	**Allele expressed^a^**
Fetus 5	Eye, kidney, liver, lung, brain, cartilage, muscle, heart	G
Fetus 7	Spleen, cotyledon, muscle, heart, testis, cartilage, lung, kidney, pancreas, liver, muscle	G
Fetus 9	Heart, pituitary, eye, cartilage, intestine, cotyledon, liver, kidney, muscle, pancreas, mammary gland, lung, spleen	A
Fetus 16	Brain, cotyledon, cartilage, muscle, mammary gland, eye, liver, intestine, spleen, kidney, lung	G
Dam 1	Ovary	G
Dam 6	Kidney, heart, caruncle, lung, ovary	A
Dam 9	Spleen, caruncle, liver, lung, ovary, oocyte, mammary gland, pancreas, endometrium	G

^a^expression of SNP alleles at position 1889 based on the numbering in GenBank accession number AY849926

Zhang et al. [6], using the single-strand conformation polymorphism method and a wide range of tissues obtained from two newborn calves, reported that bovine *H19 *is imprinted, with expression of the maternal allele. However, the authors observed low levels of expression of the paternal allele (leaky expression) in some samples. In this study, we analyzed the expression status of *H19 *in four fetuses and three adult cows and found it to be monoallelically expressed. *H19 *is imprinted and maternally expressed in cattle like its orthologues in human [25], mouse [28], and sheep [29].

## Conclusion

In this study we showed that the bovine *SLC38A4 *gene is biallelically expressed in all fetal and adult tissues examined, in contrast to the mouse gene which is paternally expressed in most fetal tissues [11]. In previous studies, we reported that the bovine *COPG2*, *DCN*, and *SDHD *[9] and the ovine *SDHD *and *COPG2 *genes [27] were not imprinted in contrast to their corresponding genes in human or mouse. Similarly, the imprinting of *SLC38A4 *appears to be species-specific. Okamura and Ito [30] found that species-specific imprinting could be explained by structural elements like tandem repeat or transposon insertions that affect allele-specific expression. We found bovine *NAP1L5 *to be imprinted and paternally expressed, like the mouse gene. However, we showed that the tissue distribution of *NAP1L5 *expression in cattle is different from that of the mouse. It is of interest that three of four examined genes examined in this study appeared to be conserved, including *NNAT*. This gene is located in an imprinted microdomain within the intron of the *BLCAP *gene. However, the tissue distribution of expression of the conserved genes differed between mouse and cow.

## Methods

### Nucleic acid preparation from tissues

Tissues from 20 cattle fetuses and their dams were obtained from an abattoir. Fetal tissues included liver, kidney, brain, lung, heart, pituitary, skeletal muscle, eye, cartilage, pancreas, ovary, mammary gland, intestine, spleen, testis, and cotyledon. Tissues recovered from the dams were endometrium, ovary, heart, kidney, lung, spleen, oocytes, skeletal muscle, pancreas, liver, and caruncle. After dissection, tissues were immediately chilled on ice and submerged in an appropriate volume of RNALater RNA stabilization reagent (Qiagen, Valencia, CA). DNA was extracted from tissues by grinding them in liquid nitrogen and using the AquaPure Genomic DNA kit (Bio-Rad). Total RNA was isolated within two hours after tissue sampling using the RNeasy kit (Qiagen). The RNA was treated with DNase I using the RNase-Free DNase Set (Qiagen) as recommended by the manufacturer except for extending the incubation to 60–70 min from 15 min. Because of the high sensitivity of RT-PCR reactions, RNA samples were subjected to an additional round of DNase I digestion using Amplification Grade DNase I (Sigma, St. Louis, MO). The DNase I digestion conditions were as recommended by the manufacturer except for a 60 min incubation at room temperature instead of 15 min.

### Polymorphism detection

For the *SLC38A4*, *NNAT*, and *NAP1L5 *genes, in silico searches were performed to identify nucleotide dissimilarities between coding sequences of these genes and cow ESTs deposited in the Genbank database [31] using the basic local alignment search tool (BLAST). Positions that showed nucleotide differences were further examined for single nucleotide polymorphism (SNP) validation. Then, primers were designed in each gene to amplify candidate SNP regions using a pooled RNA sequence-based approach. RNA pools were constructed from 4 to10 different tissues obtained from 4 to10 different animals and amplified with unlabeled primers. Amplification of cDNA was performed as previously described [9].

Reverse transcriptase (RT) PCR products were purified and sequenced according to standard procedures (Applied Biosystems, Foster City, CA). Data were analyzed using Applied Biosystems' Sequencing Analysis (version 5.0). SNPs were identified by visually inspecting each base in all sequencing traces from the pooled RNA samples. Confirmation of SNPs was carried out by individually amplifying and sequencing genomic DNA samples from the fetuses and dams that composed the pooled RNA samples.

The PCR conditions for amplifying genomic DNA from fetuses and dams were as follows: 95°C for 5 min; 30 cycles of 94°C for 45 s, touchdown annealing from 63°C – 50°C for 45 s (-2°C/3 cycles); and a final extension at 72°C for 7 min. The sizes of PCR products were estimated on a 1% agarose gel. The products were purified from agarose gel using the GFX™ PCR DNA Purification Kit (Amersham Biosciences). Table 1 shows primer sequences, PCR product sizes, and the total number of individuals examined for each gene. For *H19*, primers JY511 and JY318 designed in exon 5 were used to amplify genomic DNA from 40 animals to identify heterozygous individuals for the A/G SNP reported previously by Zhang et al. [6].

### Primer design and RT-PCR

For genes *SLC38A4*, *NNAT*, and *H19*, primers (Table 1) were designed to amplify fragments spanning more than one exon to exclude the possibility of mistyping due to genomic DNA contamination in the RT-PCR reactions. Primers SLC-F and SLC-R were used to amplify 354 bp from *SLC38A4 *cDNA (GenBank accession number NW_391237). Primers NNAT-FL/NNAT-R and NNAT-FS/NNAT-R were used to amplify the large transcript (α) and the small transcript (β) of the *NNAT *gene (GenBank accession number NW_275903) respectively. Primers H19F and H19R were used to amplify 580 bp from *H19 *(GenBank accession number AY849926). For the intron-less gene *NAP1L5 *(GenBank accession number XM_585294), primers NAP1L5-F and NAP1L5-R were used to amplify 730 bp of the bovine gene from genomic DNA and cDNA. Primers b-actin F/b-actin R were used to amplify 191 bp from the housekeeping gene b-actin (GenBank accession number NM_173979) cDNA. RT-PCR products of the two transcripts of *NNAT *and the transcript of *NAP1L5 *were mixed with RT-PCR products of b-actin, amplified from the same tissues, and separated on a 2.5% agarose gel.

SNPs identified in heterozygous individuals were employed to distinguish between monoallelic and biallelic expression. The principle is that an imprinted gene would exhibit hemizygosity (monoallelic expression), whereas a biallelically expressed gene (not imprinted) would exhibit heterozygosity at the SNP. Dams of heterozygous individuals were genotyped to identify parental origin in cases of monoallelic expression.

### Primer extension assay

To remove primers and unincorporated dNTPs, PCR and RT-PCR products were purified from agarose gel using the GFX™ PCR DNA purification Kit (Amersham Biosciences). Primer extension reactions were prepared in a total volume of 10 μl containing 1 μl of purified product, 5 μl SnaPshot Kit (Applied Biosystems), 0.02 μM extension primer, and 1 μl deionized water. The primer extension reactions were subjected to 25 cycles of 96°C for 10 sec, 50°C for 5 sec, and 60°C for 30 sec. In a post-extension treatment, reactions were treated with 1 unit of shrimp alkaline phosphatase at 37°C for 1 hour followed by deactivation of the enzyme at 75°C for 15 min. Primer sequences and sizes of primer extension products are shown in Table 1. Samples were electrophoresed on a 3700 DNA sequencer (PE Applied Biosystems), and data were analyzed using Genescan Analyzer version 2.5 software (PE Applied Biosystems). Monoallelically expressed genes would display only one peak, while biallelically expressed genes would display two peaks corresponding to two alleles of the SNP.

## Authors' contributions

HK designed the study and wrote the manuscript. IZ isolated the RNA, designed the primers, carried out the PCR and RT-PCR amplifications, and conducted the expression analysis. Both authors read and approved the final manuscript.

## References

[B1] Catalogue of Imprinted Genes Database. http://www.otago.ac.nz/IGC.

[B2] Killian JK, Nolan CM, Wylie AA, Li T, Vu TH, Hoffman AR, Jirtle RL (2001). Divergent evolution in M6P/IGF2R imprinting from the Jurassic to the Quaternary. Hum Mol Genet.

[B3] Dindot SV, Kent KC, Evers B, Loskutoff N, Womack J (2004). Conservation of genomic imprinting at the XIST, IGF2, and GTL2 loci in the bovine. Mamm Genome.

[B4] Kim J, Bergmann A, Lucas S, Stone R, Stubbs L (2004). Lineage-specific imprinting and evolution of the zinc-finger gene ZIM2. Genomics.

[B5] Khatib H (2004). Imprinting of *Nesp55 *in cattle. Mamm Genome.

[B6] Zhang S, Kubota C, Yang L, Zhang Y, Page R, O'Neill M, Yang X, Tian XC (2004). Genomic imprinting of H19 in naturally reproduced and cloned cattle. Biol Reprod.

[B7] Ruddock NT, Wilson KJ, Cooney MA, Korfiatis NA, Tecirlioglu RT, French AJ (2004). Analysis of imprinted messenger RNA expression during bovine preimplantation development. Biol Reprod.

[B8] Morison IM, Ramsay JP, Spencer HG (2005). A census of mammalian imprinting. Trends Genet.

[B9] Khatib H (2005). The COPG2, DCN, and SDHD genes are biallelically expressed in cattle. Mamm Genome.

[B10] Sugawara M, Nakanishi T, Fei YJ, Martindale RG, Ganapathy ME, Leibach FH, Ganapathy V (2000). Structure and function of SLC38A4, a new subtype of amino acid transport system A, primarily expressed in the liver and skeletal muscle. Biochim Biophys Acta.

[B11] Mizuno Y, Sotomaru Y, Katsuzawa Y, Kono T, Meguro M, Oshimura M, Kawai J, Tomaru Y, Kiyosawa H, Nikaido I, Amanuma H, Hayashizaki Y, Okazaki Y (2002). Asb4, Ata3, and Dcn are novel imprinted genes identified by high-throughput screening using RIKEN cDNA microarray. Biochem Biophys Res Commun.

[B12] Smith RJ, Dean W, Konfortova G, Kelsey G (2003). Identification of novel imprinted genes in a genome-wide screen for maternal methylation. Genome Res.

[B13] Joseph R, Dou D, Tsang W (1994). Molecular cloning of a novel mRNA (neuronatin) that is highly expressed in neonatal mammalian brain. Biochem Biophys Res Commun.

[B14] Joseph R, Dou D, Tsang W (1995). Neuronatin mRNA: alternatively spliced forms of a novel brain-specific mammalian developmental gene. Brain Res.

[B15] Dou D, Joseph R (1996). Structure and organization of the human neuronatin gene. Genomics.

[B16] Evans HK, Wylie AA, Murphy SK, Jirtle RL (2001). The neuronatin gene resides in a "micro-imprinted" domain on human chromosome 20q11.2. Genomics.

[B17] Kagitani F, Kuroiwa Y, Wakana S, Shiroishi T, Miyoshi N, Kobayashi S, Nishida M, Kohda T, Kaneko-Ishino T, Ishino F (1997). Peg5/Neuronatin is an imprinted gene located on sub-distal chromosome 2 in the mouse. Nucleic Acids Res.

[B18] Loyola A, Almouzni G (2004). Histone chaperones, a supporting role in the limelight. Biochim Biophys Acta.

[B19] Pachnis V, Belayew A, Tilghman SM (1984). Locus unlinked to alpha-fetoprotein under the control of the murine raf and Rif genes. Proc Natl Acad Sci.

[B20] Goshen R, Rachmilewitz J, Schneider T, de-Groot N, Ariel I, Palti Z, Hochberg AA (1993). The expression of the H-19 and IGF-2 genes during human embryogenesis and placental development. Mol Reprod Dev.

[B21] Lee RS, Depree KM, Davey HW (2002). The sheep (Ovis aries) H19 gene: genomic structure and expression patterns, from the preimplantation embryo to adulthood. Gene.

[B22] Yan H, Yuan W, Velculescu VE, Vogelstein B, Kinzler KW (2002). Allelic variation in human gene expression. Science.

[B23] Bray NJ, Buckland PR, Owen MJ, O'Donovan MC (2003). Cis-acting variation in the expression of a high proportion of genes in human brain. Hum Genet.

[B24] Khatib H (2005). Characterization and analysis of the imprinting status of the ovine *SDHD *and *COPG2 *genes. Anim Genet.

[B25] Rachmilewitz J, Goshen R, Ariel I, Schneider T, de Groot N, Hochberg A (1992). Parental imprinting of the human H19 gene. FEBS Lett.

[B26] Zhang Y, Tycko B (1992). Monoallelic expression of the human H19 gene. Nat Genet.

[B27] Zhang Y, Shields T, Crenshaw T, Hao Y, Moulton T, Tycko B (1993). Imprinting of human H19: allele-specific CpG methylation, loss of the active allele in Wilms tumor, and potential for somatic allele switching. Am J Hum Genet.

[B28] Bartolomei MS, Zemel S, Tilghman SM (1991). Parental imprinting of the mouse H19 gene. Nature.

[B29] Hagemann LJ, Peterson AJ, Weilert LL, Lee RS, Tervit HR (1998). In vitro and early in vivo development of sheep gynogenones and putative androgenones. Mol Reprod Dev.

[B30] Okamura K, Ito T (2006). Lessons from comparative analysis of species-specific imprinted genes. Cytogenet Genome Res.

[B31] Genbank Database. http://www.ncbi.nlm.nih.gov.

